# Post-COVID syndrome’s impact on sex life – results from a cross-sectional study

**DOI:** 10.1186/s12889-026-30045-0

**Published:** 2026-10-22

**Authors:** Dominik Schröder, Tim Schmachtenberg, Stephanie Heinemann, Christina Müllenmeister, Alexandra Dopfer-Jablonka, Eva Hummers, Eva Maria Noack, Marie Mikuteit, Jacqueline Niewolik, Sandra Steffens, Frank Klawonn, Andrea Stölting, Christine Happle, Frank Müller

**Affiliations:** 1 Department of General Practice, University Medical Center Göttingen, Humboldtallee 38, Göttingen, 37073 Germany; 2 Department of Rheumatology and Immunology, Hannover Medical School, Hannover, Germany; 3 Department of Geriatrics, University Medical Center Göttingen, Göttingen, Germany; 4 German Center for Infection Research (DZIF), partner site Hannover-Braunschweig, Braunschweig, Germany; 5 Department of Dermatology and Allergy, Hannover Medical School, Hannover, Germany; 6 Department of Medical Education, Hannover Medical School, Hannover, Germany; 7 Institute for General Practice and Palliative Care, Hannover Medical School, Hannover, Germany; 8 Department of Computer Science, Ostfalia University of Applied Sciences, Wolfenbüttel, Germany; 9 Biostatistics Group, Helmholtz Center for Infection Research, Braunschweig, Germany; 10 German Center for Infection Research, RESIST, Hannover-Braunschweig, Germany; 11 German Center for Lung Research, DZL-BREATH, Hannover, Germany; 12 Department of Family Medicine, Michigan State University, Grand Rapids, MI US

**Keywords:** PCS, Social participation, Sex life, Sexuality, Sexual dysfunction, Long COVID

## Abstract

**Background:**

Having a satisfying sexual life is important to many people and impairment of sexual function is often perceived as a burden. This study investigates the self-perceived impairments to sex life in adults with Post-COVID-19 syndrome (PCS) and factors that are associated with impaired sex life.

**Methods:**

A cross-sectional online survey of people with PCS and without PCS was conducted in Northern Germany. Sex life impairments were assessed using item 8 (frequency and quality of sex life) of the Index for the Assessment of Health Impairments (IMET), scored from 0 (no impairment) to 10 (no activity possible). Scores were analyzed as a continuous outcome because no validated cut-offs have been established for this individual item. Linear regression models were used to adjust for sociodemographic factors as well as medical and mental health conditions. Sample weighting for age and gender was applied in sensitivity analyses to adjust for a representative sample of people with PCS.

**Results:**

A total of 892 participants were included, with 67.8% (n = 605) reporting PCS. Persons with PCS reported significantly higher sex life impairments (mean score 4.62) compared to those without PCS (mean score 2.58, p < 0.001). The regression model confirmed that PCS was associated with sex life impairments (β = 1.16, p < 0.001). No significant correlation was found between sex life impairments and the symptom duration symptoms or the respondents´ gender.

**Conclusions:**

Impaired sex life is a significant concern for PCS patients, that warrants attention from healthcare providers. Our findings highlight the importance of addressing sex life impairments in the care of people with PCS.

**Supplementary Information:**

The online version contains supplementary material available at https://doi.org/10.1186/s12889-026-30045-0.

## Background

Beginning in 2020, severe acute respiratory syndrome coronavirus type 2 (SARS-CoV-2) caused a global pandemic that was associated with both acute infections and long-term health impairments. A proportion of people infected with SARS-CoV-2 experience persistent or novel symptoms that persist four weeks or longer after post infection, commonly referred to as Post-COVID-19 Syndrome (PCS) [1, 2]. In the second quarter of 2023, 246.066 out of 73 million publicly insured people in Germany received ambulatory care due to PCS [3].

PCS is considered a multisystem condition, and patients may experience a variety of symptoms including chronic fatigue, cognitive impairment, shortness of breath, headache, joint and muscle pain, vertigo and dizziness, symptoms of anxiety or depression, and many more [1, 4–8].

So far, no effective treatment for PCS is known. Healthcare systems are struggling to provide care for large numbers of people with PCS and many affected patients perceive their medical care as inadequate [9, 10]. People with PCS show decreased social functioning and an impairment in health-related quality in life [9, 11].

Impaired sex life following an acute SARS-CoV-2 infection has been reported in several studies [12, 13], mainly as erectile dysfunction among males [14]. However, evidence regarding sexual impairment in people with PCS is scarce: Authors suggested that the occurrence of erectile dysfunction in men with PCS can be as high as 45%, but symptoms may decrease over time [15]. Another study reported the frequency of PCS-associated sexual dysfunction to be 36% in men and 28% in women [16]. Sexual dysfunction in PCS has been associated with emotional distress and other persistent symptoms [16], although their role in explaining impaired sexual function remains uncertain [17]. Possible physiological mechanisms include penile endothelial dysfunction, suggested by a small tissue study, and hormonal changes, with persistently low testosterone levels reported in some men after hospitalization for COVID-19 [18, 19]. However, the relative contributions of these mechanisms and general PCS symptoms to sexual impairment remain unclear. Other studies on people with chronic conditions with similar features such as PCS, such as fibromyalgia or Sjögren’s syndrome described considerable impairment of patient’s sex lives, that also impacted their overall quality of life [20, 21]. However, evidence on broader self-perceived impairments in sex life among people with PCS remains limited.

In this study, we investigate the following hypotheses:

1. More people with PCS report sex life impairments compared to those without PCS.
2. In persons with PCS, sex life impairment severity differs depending on the time elapsed since the index SARS-CoV-2 infection.

## Methods

This study investigates sex life impairments in persons with and without PCS in Germany using cross-sectional data from the DEFEAT-Corona cohort [22]. Classification as PCS was based on self-reported persistent or new symptoms four weeks after a test-confirmed SARS-CoV-2 infection, as detailed in the section “Primary exposure variable.” For this analysis, we used data from the baseline survey that participants completed between September 17th, 2021, and September 09th, 2022. Results are reported following the STROBE guidelines [23]. The study protocol and a data protection plan were approved by the responsible research ethics boards of Hannover Medical School (9948_BO_K_2021) and University Medical Center Göttingen (29/3/21). The study is registered in the German Register for Clinical Trials (DRKS00026007 | registered on September 9th, 2022).

### Recruitment

The survey, hosted on the project website (defeat-corona.de), was made accessible to participants beginning in September 2021. To enhance the survey´s outreach and visibility, QR codes directing individuals to the project website were prominently featured on posters and flyers distributed across diverse public and communal spaces, including libraries, town halls, and hospitals in the German Federal State of Lower Saxony. Furthermore, information material was sent to 400 randomly selected general practices, internal medicine practices, radiology practices, and occupational therapy practices in Lower Saxony. Although recruitment primarily focused on Lower Saxony, where the study team was based, participation was open to eligible individuals throughout Germany. PCS patient advocacy groups were contacted to share the survey link through their respective social media platforms. Recruitment materials focused on COVID-19 and its long-term consequences and did not specifically mention sex life impairments. The survey was administered using the SoSci Survey online platform (Survey GmbH, Munich, Germany). It was available only in German due to limited resources for translating, adapting, and pretesting additional language versions of the full questionnaire. We opted for an online survey method as it reduced participation barriers in our target group with considerable symptom burden. Furthermore, an online survey approach has been shown to reduce biases in responses to sensitive topics such as impairment of sex life [24]. Prior to study inclusion, participants were required to provide digital consent and confirm a minimum age of 16 years. To facilitate subsequent follow-up surveys, the study team assigned each participant a pseudonym linked to their email address in the online study portal. Further details are outlined in the study protocol [22].

### Eligibility criteria

Eligible participants for data analysis needed (a) to be between 20 and 60 years old, (b) currently residing in Germany, and (c) proficient in the German language. The age limit was chosen because there were only enough cases in our sample up to this age to weight by gender and age.

### Sample size

The specific research question concerning PCS-related sex life impairments emerged during the ongoing DEFEAT-Corona study and was not prespecified at study initiation in 2021. Accordingly, no outcome-specific a priori sample size calculation was performed for this exploratory analysis. The convenience sample comprised all respondents who participated during the specified data collection period, met the eligibility criteria, and provided valid outcome data.

### Outcome

Maintaining intimate relationships including those a of sexual nature is considered a key part of human nature and is as such acknowledged in the WHO’s International Classification of Functioning, Disability and Health (ICF) [21]. For this study, we used the instrument “Index for the Assessment of Health Impairments (IMET)” which contains a particular item inquiring about the ability to maintain a satisfying sexuality or intimate sexual relationship form [25] that closely adheres to the WHO-ICF. The IMET was initially developed to assess social participation and efficacy of rehabilitation interventions in people with chronic conditions, such as inflammatory bowel disease [26, 27], mental health conditions [28] neurological conditions [29, 30], or diabetes [31]. It has been validated in various settings and showed strong internal reliability (Cronbach’s alpha 0.90 [32]). The IMET was used in rehabilitation research as well as investigations on COVID-19 [33–35] and PCS [11].

The IMET consists of nine items rated from 0 (no impairment) to 10 (no activity possible), with higher scores indicating greater impairment [20]. Item 8 was used as the outcome measure: *“Sex life (this area refers to both frequency and quality of sex life)”* (translated from German). The survey instructions were phrased generally, without reference to illness or PCS, making the item applicable to participants without a previous SARS-CoV-2 infection. Participants provided a single overall rating; frequency and quality were not assessed separately.

### Primary exposure variable

Participants were categorized in two groups: (1) “PCS” when they stated to have persistent or new symptoms four weeks after a SARS-CoV-2 infection confirmed by an antibody test, PCR or positive rapid antigen test and (2) “no PCS” when participants reported no previous or current SARS-CoV-2 infection at the time of completing the questionnaire. The four-week threshold was based on the broader definition of long COVID described in the NICE guideline available at study initiation [36]. PCS classification was based on self-report, and participants were not provided with a predefined list of qualifying symptoms.

### Covariates

#### Sociodemographic variables

Covariates included gender (“To which gender identity do you most identify?”, response options: male, female, or gender-diverse), age (in years), highest school-leaving qualification (low: no or lower secondary school qualification; medium: intermediate secondary school qualification; high: university entrance qualification), marital status, employment status (full-time, part-time, or not currently working, e.g. unemployed, retired, in training, or on parental leave), and parenting status (having children under 18 years old in the household). Two participants identified as diverse and were included in the study cohort. They were excluded from the present analyses because of the small subgroup size. The education item did not assess vocational qualifications or university degrees.

#### Medical variables

Participants were asked about comorbidities. For this analysis, we grouped cardiac conditions (hypertension, heart failure, coronary heart disease, or cardiac arrhythmia) and chronic inflammatory/autoimmune conditions (ulcerous colitis, chronic hepatitis, psoriasis, neurodermatitis, Graves’ diseases & Hashimoto’s thyroiditis, rheumatoid arthritis, or rheumatic disease). Additionally, we used the German versions of the 9-item Patient Health Questionnaire (PHQ-9) [37] and the 7-item Generalized Anxiety Disorder Scale (GAD-7) [38] to assess depression and anxiety symptoms. We utilized previously suggested cut-off points for both instruments, with PHQ-9 sum scores above 14 indicating moderate to severe depression symptoms and GAD-7 sum scores above 9 indicating moderate to severe anxiety symptoms [37, 38]. Neither PHQ-9 nor GAD-7 scores were used for PCS classification, which was based on self-reported persistent or new symptoms four weeks after infection, without requiring a predefined set of qualifying symptoms. COVID-19 vaccination status was self-reported and defined as receipt of at least one vaccine dose at the time of the baseline survey, irrespective of vaccine type. Vaccination before COVID-19 was defined as receipt of the first vaccine dose before the reported onset of COVID-19 symptoms.

### Efforts for bias reduction

Risk of bias was addressed in various ways. To exclude information from respondents filling out the questionnaire repeatedly, we checked for pseudonym repetitions and kept surveys with the most completed items. Furthermore, participants with missing values in the sex life impairment score or the covariates included in the regression models were excluded from the analytical sample. No imputation was performed. As our study could have been prone to self-selection bias [39], we conducted additional sensitivity analyses in which we applied sample weighting according to age and gender distribution of the persons with PCS reported in current health insurance data (*n* = 246,066 people with PCS [3]) covering all approximately 73 million people insured through German statutory health insurance [40]. Within the PCS group, participants were assigned to eight strata defined by gender (men/women) and age group (20–29, 30–39, 40–49, and 50–59 years). The corresponding proportions from the health insurance report [3] were used as weighting targets. Individual weights were calculated by raking using the R package anesrake [41] with default settings, including a weight cap of 5, and applied to the multivariable linear regression in the sensitivity analysis.

### Data analyses

We used descriptive statistics including frequency of observations and proportions for categorical variables. Mean (standard deviations) or median (Q25-Q75) was used for continuous variables. Descriptive statistics were stratified for PCS status to characterize our sample.

The hypothesis that PCS was associated with impaired sex life was quantified using two approaches: First, the bivariate Wilcoxon rank sum test was used to compare sex life impairment scoring between participants with PCS and without previous SARS-CoV-2 infections. Second, we performed linear regression models with sex life impairment scoring as dependent and PCS status as an independent variable. The regression models were adjusted for gender, age, school education, marital status, employment status, parenting status, comorbidities (heart disease and autoimmune disease), depression symptoms, and anxiety symptoms. Interaction terms between (a) gender and PCS and (b) gender and depressive symptoms (PHQ-9) were included, because the literature suggests differences in the severity of PCS symptoms and depressive symptoms between genders [42–46].

To test the hypothesis that sex life impairments are associated with the symptom duration, bivariate and multivariable analyses were conducted including a Pearson correlation between sex life impairment scores and days since the first COVID-19 symptoms in PCS participants. Additionally, days since the first COVID-19 symptoms (of the index episode) were categorized (< 100 days, 100–199 days, 200–300 days, and > 300 days) and tested for differences in sex life impairment scores between the categories using the Kruskal-Wallis test.

A multivariable linear regression was conducted using the sex life impairment score as an dependent variable, and PCS status as predictor. The regression model was adjusted for gender, age, school education, marriage status, employment status, parenting status, comorbidities (heart disease and autoimmune disease), depression symptoms (including an interaction with gender), anxiety symptoms, and days since the onset of the first COVID-19 symptoms. Pre-pandemic standard values of the German population for sex life impairment item scores were obtained from Staupendahl [47]. Using the published summary statistics for the reference sample and the corresponding statistics from our PCS cohort, we calculated Hedges’ g with corresponding 95% confidence intervals to quantify the standardized differences between the two independent groups. Effect size magnitudes of 0.2, 0.5, and 0.8 were interpreted as small, medium, and large, respectively [48]. Regression and correlation coefficients with corresponding 95% confidence intervals and p-values were reported. P-values < 0.05 were considered statistically significant. As a goodness of fit measure for the linear regression models, R^2^ values were reported. All statistical analyses and figures were conducted using the software R (Version 4.1.3).

## Results

### Sample description

**Fig. 1:**
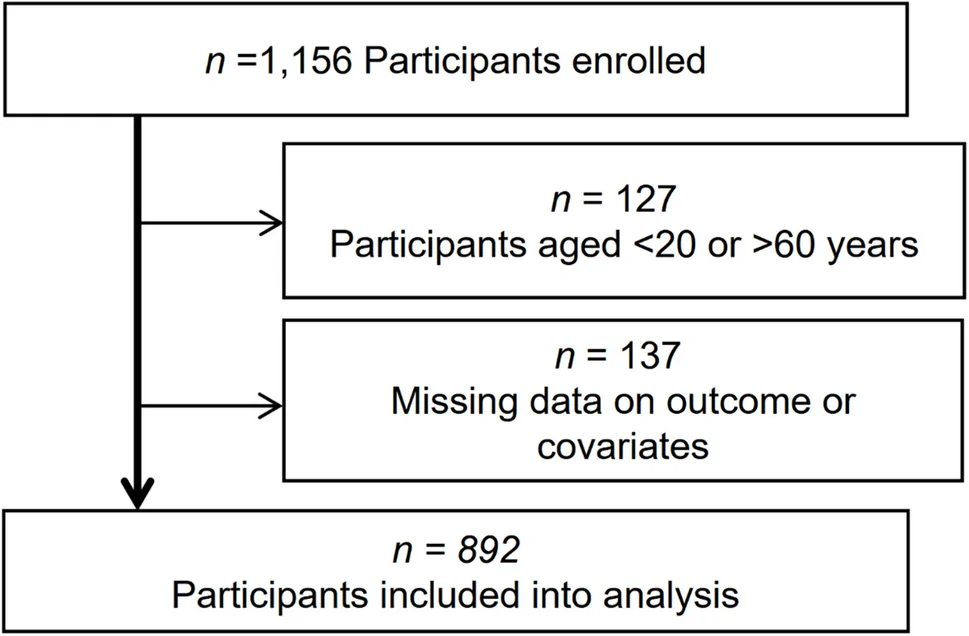
Flowchart of participant inclusion

After applying exclusion criteria, *n* = 892 subjects were included in the analyses (Fig. 1).

The mean age of included participants was 42.8 years (SD 10.9), and 67.8% (*n* = 635) had PCS. Most participants were women (*n* = 695, 77.9%), had a high school diploma (*n* = 613, 68.7%), and were working full-time (*n* = 479, 53.7%). Moderate to severe anxiety symptoms (GAD-7 scores > 9) were reported by 21.3% of the participants and 15.5% indicated moderate to severe depression symptoms (PHQ-9 scores > 14). All participant characteristics stratified for PCS status are presented in Table 1.

**Table 1:** Participant characteristics

	All (*N* = 892)	PCS (*N* = 605)	no PCS (*N* = 287)
Gender			
Men	197 (22.1)	107 (17.7)	90 (31.4)
Women	695 (77.9)	498 (82.3)	197 (68.6)
Age in years, mean (SD)	42.8 (10.9)	42.4 (10.2)	43.6 (12.1)
< 30 years	137 (15.4)	88 (14.5)	49 (17.1)
30–39 years	210 (23.5)	145 (24.0)	65 (22.6)
40–49 years	252 (28.3)	190 (31.4)	62 (21.6)
50–60 years	293 (32.8)	182 (30.1)	111 (38.7)
School education			
Low	25 (2.8)	20 (3.3)	5 (1.7)
Middle	254 (28.5)	201 (33.2)	53 (18.5)
high	613 (68.7)	384 (63.5)	229 (79.8)
Employment status			
Full-time	479 (53.7)	317 (52.4)	162 (56.4)
Part-time	276 (30.9)	198 (32.7)	78 (27.2)
Not currently working	137 (15.4)	90 (14.9)	47 (16.4)
Married			
yes	501 (56.2)	346 (57.2)	155 (54.0)
Parenting tasks			
yes	286 (32.1)	200 (33.1)	86 (30.0)
Heart disease			
yes	154 (17.3)	105 (17.4)	49 (17.1)
Autoimmune disease			
yes	382 (42.8)	271 (44.8)	111 (38.7)
Vaccinated against COVID-19			
yes	838 (93.9)	574 (94.9)	264 (92.0)
before COVID-19	N/A	231 (38.2)	N/A
Hospitalized for COVID-19			
yes	N/A	43 (7.1)	N/A
GAD-7, mean (SD)	6.05 (4.9)	6.5 (4.9)	5.1 (4.7)
sum score > 9	190 (21.3)	142 (23.5)	48 (16.7)
PHQ-9, mean (SD)	9.6 (5.4)	10.9 (4.9)	6.9 (5.5)
sum score > 14	163 (18.3)	134 (22.1)	29 (10.1)
Time since onset of COVID-19 in days, mean (SD)	N/A	287.9 (181.7)	N/A

The “no PCS” group comprised participants reporting no previous or current SARS-CoV-2 infection. Vaccinated indicates receipt of at least one COVID-19 vaccine dose by the baseline survey, irrespective of vaccine type. Infection-related characteristics are presented only for the PCS group. In the “All” column, N/A indicates that no overall summary is presented; in the “no PCS” column, it indicates that the characteristic is not applicable

Data are presented as n (%) unless otherwise stated

*PCS* post-COVID syndrome, *SD* standard deviation, *GAD-7* Generalized Anxiety Disorder-7, *PHQ-9* Patient Health Questionnaire-9

### Sex life impairments in PCS and non-PCS

The mean item score for sex life impairments was 3.96 (median = 3) with a standard deviation (SD) of 3.58 (Q25-Q75 = 0–7). In bivariate analyses, people with PCS reported significantly higher sex life impairments than those without PCS (t(890) = 8.21, *p* < 0.001): In the PCS group, the mean item score was 4.62 (median = 5) with an SD of 3.47 (Q25-Q75 = 1–8) while in the group without previous SARS-CoV-2 infection the mean item score was 2.58 (median = 1) with a SD of 3.40 (Q25-Q75 = 0–5). A difference between men (M = 4.35, SD = 3.42) and women (M = 4.67, SD = 3.49) PCS persons was not observed (t(603) = 0.86, *p* = 0.39). In multivariable analysis adjusting for sociodemographic factors and mental health, PCS continued to be associated with higher sex life impairments (β = 1.16, t(876) = 4.19, *p* < 0.001). Differences between men and women participants could not be observed in multivariable analysis (β = 0.09, t(876) = 0.18, *p* = 0.94, Table 2).

**Table 2:** Regression analyses on sex life impairments

	Sex life impairments Scale 0–10	
	β (95% CI)	*p*
Gender		
Men	0.04 (-0.97; 1.04)	0.94
Women	- (ref)	
Age (years, continuous)	0.03 (0.01; 0.05)	0.01
School education		
Low	- (ref)	
Middle	-0.27 (-1.60; 1.07)	0.70
High	-0.24 (-1.55; 1.08)	0.72
Employment status		
Full-time	- (ref)	
Part-time	0.20 (-0.31; 0.71)	0.44
Not currently working	1.00 (0.38; 1.62)	0.001
Married		
yes	-0.39 (-0.87; 0.09)	0.11
Parenting tasks		
yes	0.57 (0.08; 1.06)	0.02
Heart disease		
yes	0.15 (-0.44; 0.74)	0.63
Autoimmune disease		
yes	0.03 (-0.40; 0.47)	0.88
PCS		
yes	1.16 (0.59; 1.73)	< 0.001
Anxiety; GAD-7 (score)	-0.05 (-0.12; 0.02)	0.16
Depression; PHQ-9 (score)	0.27 (0.20; 0.34)	< 0.001
Interactions		
PCS* gender (men)	-0.59 (-1.75; 0.56)	0.31
gender (men)*PHQ-9 (score)	0.02 (-0.07; 0.12)	0.65
R^2^	0.21	

*GAD-7* Generalized Anxiety Disorder-7, *PHQ-9* Patient Health Questionnaire-9, *PCS* Post-COVID Syndrome

**Fig. 2:**
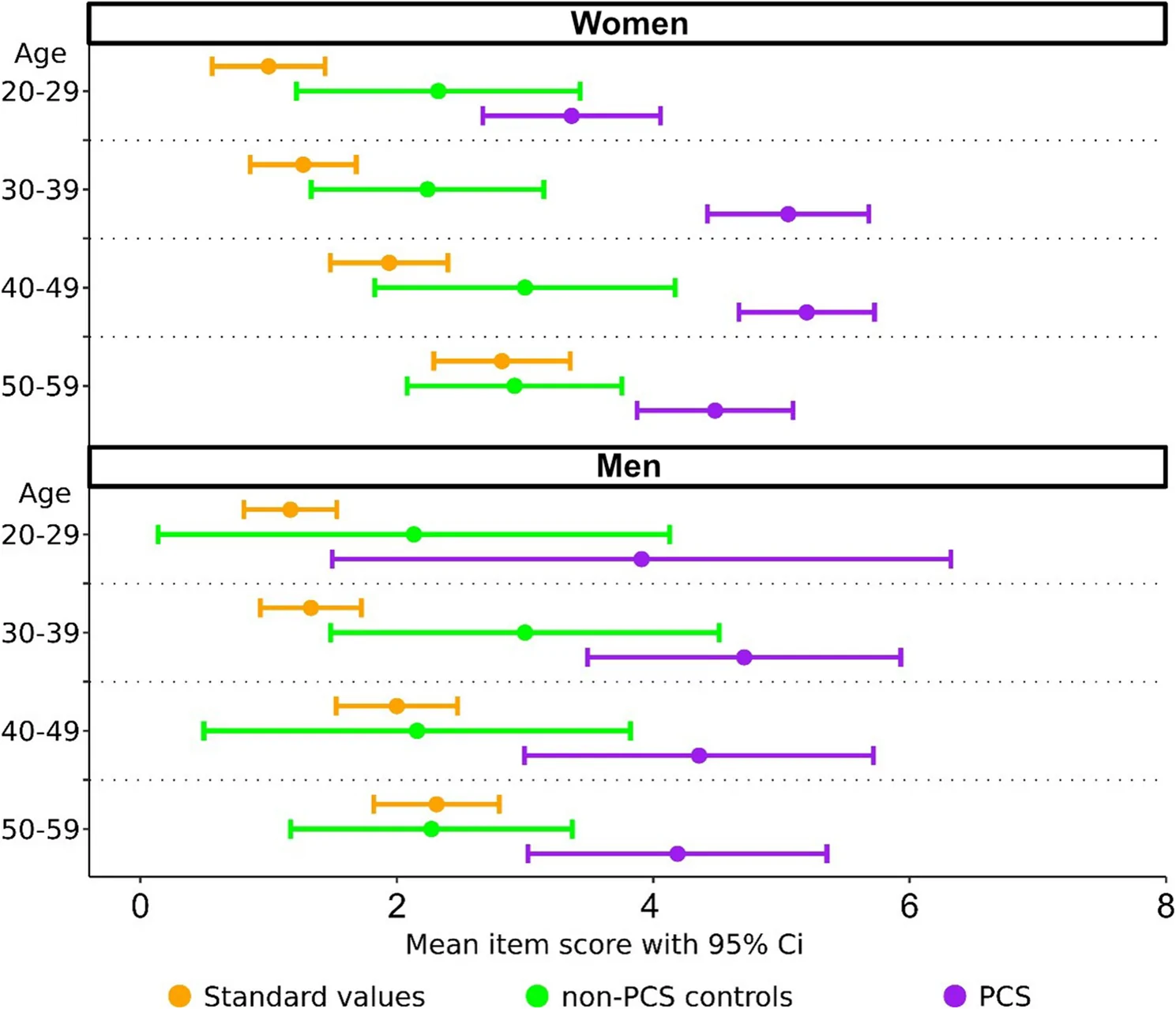
IMET item 8 on sex life stratified for age and gender. Higher item scores indicate stronger sex life impairments

Hedges g showed moderate (> 0.5) and large (> 0.8) sizes when comparing persons with PCS with pre-pandemic standard values stratified by gender and age (Table 3). Unadjusted sex life impairments among people with PCS and controls without PCS as well as pre-pandemic standard values for this item [47] stratified for age and gender are shown in Fig. 2.

**Table 3:** Hedges’ g between PCS and pre-pandemic standard values (higher effect sizes indicate stronger impairments in PCS group compared to pre-pandemic standard values)

Group	Hedges’ g	95% CI
Men 20–29	1.26	0.61–1.92
Men 30–39	1.34	0.92–1.77
Men 40–49	0.81	0.40–1.23
Men 50–60	0.40	0.04–0.76
Women 20–29	1.01	0.72–1.30
Women 30–39	1.28	1.02–1.55
Women 40–49	0.99	0.76–1.22
Women 50–59	0.62	0.40–0.85

### Factors associated with sex life impairments in individuals with PCS

No significant correlation was found between sex life impairments and days since the onset of preceding SARS-CoV-2 infections (r(605) = 0.02, *p* = 0.69). Also, when categorizing individuals according to the duration of their PCS symptoms, no significant difference in sex life impairments was found (χ^2^(3, *n* = 605) = 1.27, *p* = 0.74). In multivariable analysis adjusted for sociodemographic factors, comorbidities, and mental health, PCS duration showed no association with sex life impairments (*p* = 0.66, Table 4). While age and parenting tasks were not associated with particularly strong sex life impairments in people with PCS, not currently working and depression were - just as in the analyses for the entire cohort of respondents - linked to significantly worse sex life scorings in people with PCS (p 0.02 and *p* < 0.001, Table 4).

**Table 4:** Regression analyses on sex life impairments in persons with PCS

*N* = 605	Sex life impairments Scale 0–10		
	β (95% CI)		*p*
Gender			
Men	-0.40 (-1.12; 0.33)		0.28
Women	- (ref)		
Age (years, continuous)	0.02 (-0.00; 0.05)		0.10
School education			
Low	- (ref)		
Middle	-0.41 (-1.95; 1.14)		0.61
High	-0.30 (-1.80; 1.26)		0.73
Employment status			
Full-time	- (ref)		
Part-time	0.20 (-0.42; 0.83)		0.52
Not currently working	0.96 (0.17; 1.74)		0.02
Married			
yes	-0.10 (-0.71; 0.51)		0.75
Parenting tasks			
yes	0.56 (-0.05; 1.17)		0.07
Heart disease			
yes	0.01 (-0.72; 0.74)		0.98
Autoimmune disease			
yes	-0.38 (-0.92; 0.16)		0.17
Anxiety; GAD-7 (score)	-0.035 (0.04; -0.11)		0.38
Depression; PHQ-9 (score)	0.26 (0.18; 0.34)		< 0.001
Symptom duration (in days)	-0.00 (-0.00; 0.00)		0.66
R^2^	0.13		

*GAD-7* Generalized Anxiety Disorder-7, *PHQ-9* Patient Health Questionnaire-9

Applying gender and age weighting to approximate representativity based on health insurance data did not change significance in our model (Supplemental Table S1).

## Discussion

Our findings supported the first hypothesis: participants with PCS reported greater sex life impairments than participants with no previous SARS-CoV-2 infection, and this association remained after adjustment for the measured covariates. The second hypothesis was not supported, as sex life impairment scores were not significantly associated with time since the onset of the index COVID-19 episode within the PCS group.

The finding that PCS is associated with an impaired sex life is not surprising: Besides a direct impact of SARS-CoV-2 infections on sexual organs [12, 13], PCS-related symptoms such as fatigue, subjective shortness of breath, palpitations, or pain may have a considerable negative effect on sexual activity and satisfaction. Furthermore, people with PCS who experience post-exertional malaise (PEM) may avoid sexual activity due to a fear of relapse [49].

We confirm the results of earlier studies on sexual impairments in PCS [14–16]. These studies mostly focused on male erectile dysfunction [15]. Our sample predominantly includes women (77.9%) extends this evidence by highlighting sex life impairments among women with PCS. Sexual difficulties have also been reported among trans and nonbinary people with PCS and, outside PCS research, among transgender people and people with variations in sex characteristics [50–52]. Given reported differences in sexual function by sexual orientation [53], future PCS studies should include more diverse samples and consider sexual orientation and sexual practices alongside gender identity and sex characteristics. Direct comparisons of our work with previous papers on sexual dysfunction in PCS are difficult as different instruments were employed, e.g., for erectile dysfunction (IIEF-5) [14] or a three-item likert scale on “*difficulties in sexual function following*” [16]. Our approach assessed participants’ perceived impairment in sex life using a single rating. However, we did not examine how participants interpreted the item or which psychological, physiological, relational, or environmental aspects they considered.

Other researchers have described that chronic conditions negatively impact sexuality [54, 55] including conditions that share similarities with PCS such as fibromyalgia or Sjögren’s Syndrome [20, 21]. This is consistent with our finding that participants with self-reported PCS reported greater sex life impairment than those without PCS. However an older study could not find differences in sexual dysfunction between females with chronic fatigue syndrome and healthy controls [56].

Participants without previous SARS-CoV-2 infection had higher mean impairment scores than the pre-pandemic reference sample in some age and gender strata, while scores were similar among those aged 50–59 years. Wide confidence intervals in some control subgroups indicate limited precision. The observed pattern may reflect sampling variability, differences in sample composition, or changes in social and psychological circumstances between the data collection periods. Pandemic-related changes may also have affected sex life among participants without previous infection [57, 58]. These possible explanations could not be examined directly in our study. Besides the impact of PCS on sex life, our multivariable analyses showed smaller, but statistically significant effects for age, currently not working, having parenting tasks, or suffering from depressive symptoms across all respondents. Age has long been described as a relevant factor in sexuality and libido of both men and women [59]. Additionally the negative implications of parenting stress, depression, and unemployment on an individual´s sex life have been described in other studies [60–62]. In our study, the associations of sex life impairments with depressive symptoms and not currently working suggest that psychological and social circumstances are relevant to understanding these impairments. PCS remained associated with higher impairment scores after adjustment for the measured sociodemographic and mental health factors, indicating that these factors did not fully account for the observed difference between groups.

When looking only at people with PCS, age and parenting tasks had no significant effect on sex life impairments. However, effect sizes in our calculations suggested that highest impairments compared to pre-pandemic controls occurred in men and women with PCS aged between 30 and 39, followed by 20 to 29-year-olds. Not currently working and higher depressive symptom scores were associated with greater sex life impairments. These findings are in line with previous findings and concepts on the negative influence of depression and working status on sexuality [63–65] and are particularly concerning, as PCS is associated with an increased frequency of unemployment [66] and depression [67].

The lack of a significant association with time since COVID-19 symptom onset does not mean that sex life impairments remain unchanged. We assessed each participant only once and could therefore not examine individual changes over time. The time since the initial illness may also differ from the duration of ongoing symptoms. Recovery may vary between individuals, and people with more persistent symptoms may have been more likely to participate. Longitudinal studies with repeated assessments over several years are needed to examine how sex life impairments change alongside other symptoms, mental health, and social circumstances. Comparisons between people with PCS, including those who meet criteria for myalgic encephalomyelitis/chronic fatigue syndrome (ME/CFS), and people without PCS could help distinguish illness-related changes from broader effects of the pandemic.

Previous research has linked sexual satisfaction to overall quality of life [68]. In this context, the higher sex life impairment scores observed in participants with PCS may be relevant to their broader well-being. However, the relationship between sex life impairments and quality of life was not examined in the present analysis. Further research is needed to assess this association specifically in people with PCS, considering other symptoms and psychosocial factors. Longitudinal studies could help clarify whether changes in sex life impairments are accompanied by changes in quality of life and how this relationship develops over time.

Currently, many guidelines for PCS management including the UK NICE guidelines [36] or German National Care Guideline [69] neither mention impaired sexuality nor provide information on symptom management or treatment. If public attention to PCS declines, sex life impairments may receive less recognition in clinical care and health policy, potentially hindering access to support. Maintaining awareness of these concerns therefore remains important beyond the initial pandemic period. Future studies should evaluate whether symptom management, psychological support, or psychosexual counselling can improve sex life in PCS, as the present study did not assess the effectiveness of interventions.

### Limitations

Our study has several limitations. While the nature of our online study with self-reported data collection may have reduced the bias towards responding in a socially expected manner with regard to the still stigmatized subject of impaired sex life, it may have been associated with a self-selection bias [39]. Recruitment through PCS patient groups may have contributed to the larger PCS group and influenced the observed associations. Although recruitment materials did not mention sex life impairments, we cannot exclude that people experiencing such impairments were more likely to participate. The smaller comparison group may also have reduced the precision of between-group estimates. We addressed this limitation partly by applying sample weighting in sensitivity analyses which confirmed our findings. Nevertheless, due to the online and self-selected nature of recruitment, our study sample cannot be considered representative of the general population, as also reflected in its descriptive characteristics. Sample weighting can only partly address this limitation, as it cannot account for unmeasured differences between participants and the general population. The German-language online survey limited participation among people with limited German language proficiency or internet access, while those avoiding public spaces during the pandemic may have been less likely to encounter printed recruitment materials. Gender identity was assessed, but information on biological sex characteristics was unavailable for this analysis. We therefore could not examine whether differences in sex life impairments were related to specific physiological characteristics. The two participants identifying as diverse were excluded solely for statistical reasons, as the subgroup was too small to obtain reliable group-specific estimates. This limits the applicability of our findings to gender-diverse individuals. Transgender, nonbinary, or intersex status, sexual orientation, and sexual practices were not assessed, so differences in sex life impairments related to these characteristics could not be examined. We did not assess sex life impairments for those under 20 or over 60 years, as data for sample weighting or standard values for these age groups were unavailable. In addition, PCS status was based on self-report and was not clinically verified. This may have resulted in misclassification, as participants’ attribution of persistent symptoms to PCS could not be independently confirmed. The binary PCS classification did not capture differences in symptom type, number, or severity, and overall PCS symptom burden was not accounted for in this analysis. Standard values for sexual impairment from the era after onset of the COVID-19 pandemic would have been desirable but was also unavailable. Pre-infection sex life impairment scores were unavailable for participants with PCS. We therefore could not determine whether their impairments were already present before infection or developed or worsened afterwards. Another limitation of our analysis lies in the operationalization to measure the impairments to sex life in a single item which did not allow further distinction of causes leading to impaired sex life quality. We addressed this by analyzing associated factors, such as social stressors and mental health and general health conditions using regression models. Although the complete IMET has been validated, we did not assess how participants interpreted the individual sex life item. The single item combines frequency and quality of sex life and does not allow these potentially distinct aspects to be examined separately.

## Conclusions

Our research indicates that impaired sexual life is a significant concern impacting a substantial proportion of people with PCS. Health care professionals taking care of patients with PCS should be aware that PCS is associated with sexual health complaints and consider discussing this actively with their patients. Our findings should inform PCS management, treatment, and rehabilitation guidelines which currently lack information and recommendations on this topic. While our study offered a first insight into impaired sex life among individuals with PCS, future studies are needed to gain a deeper understanding of psychosocial and pathophysiological factors intertwined with sexual health in PCS. Furthermore, qualitative and mixed-methods approaches may provide information about how people with PCS and their partners experience their sex lives as well as related health care needs. This knowledge is urgently needed to clarify the potential need and scope for therapeutic interventions and to ultimately improve health in PCS patients.

## Supplementary Information

Supplementary Material 1.

## Data Availability

The datasets generated and analyzed during the current study are not publicly available in accordance with the decision of the involved Research Ethics Boards but are available from the corresponding author on reasonable request within a data sharing agreement.

## Abbreviations

GAD-7: Generalized Anxiety Disorder-7
ICF: International Classification of Functioning, Disability and Health
IIEF-5: 5-item International Index of Erectile Function
IMET: Index for the Assessment of Health Impairments
ME/CFS: Myalgic encephalomyelitis/chronic fatigue syndrome
NICE: National Institute for Health and Care Excellence
PCS: Post-COVID syndrome
PEM: Post-exertional malaise
PHQ-9: Patient Health Questionnaire-9
SARS-CoV-2: Severe acute respiratory syndrome coronavirus 2
