# Supplementary material for: Post-COVID syndrome’s impact on sex life – results from a cross-sectional study
Source: BMC Public Health. 2026 Oct 22;26:3043. doi: 10.1186/s12889-026-30045-0 (PMC13693165; doi:10.1186/s12889-026-30045-0)
Supplement: Supplementary file 1 [file 12889_2026_30045_MOESM1_ESM.docx]

# Supplemental File

**Table S1:** Participants with PCS (sample weighted for age & gender)

| N=605 | Sex life impairments  Scale 0-10 | |
| --- | --- | --- |
|  | β (95% CI) | p |
| Gender |  |  |
| Male | -0.38 | 0.22 |
| Female | - (ref) |  |
| Age (per year) | 0.02 | 0.15 |
| School education |  |  |
| Low | - (ref) |  |
| Middle | 0.27 (-1.00; 1.53) | 0.68 |
| high | 0.33 (-0.93; 1.58) | 0.61 |
| **Employment status** |  |  |
| Full-time | - (ref) |  |
| Part-time | 0.26 (-0.38; 0.89) | 0.43 |
| **unemployed** | **0.88 (0.10; 1.66)** | **0.03** |
| Married |  |  |
| yes | -0.26 (-0.86; 0.34) | 0.41 |
| Parenting tasks |  |  |
| yes | 0.47 (-0.13; 1.08) | 0.12 |
| Heart disease |  |  |
| yes | -0.07 (-0.75; 0.61) | 0.85 |
| Autoimmune disease |  |  |
| yes | -0.40 (-0.95; 0.13) | 0.14 |
| Anxiety; GAD-7 (score) | -0.05 (-0.13; 0.03) | 0.25 |
| **Depression; PHQ-9 (score)** | **0.28 (0.19; 0.36)** | **<0.001** |
| PCS duration | 0.00 (-0.00; 0.00) | 0.78 |
|  |  |  |
| R^2^ | 0.15 |  |

GAD-7: Generalized Anxiety Disorder-7; PHQ-9: Patient Health Questionnaire-9
